# Toward Growing Robots: A Historical Evolution from Cellular to Plant-Inspired Robotics

**DOI:** 10.3389/frobt.2018.00016

**Published:** 2018-03-14

**Authors:** Emanuela Del Dottore, Ali Sadeghi, Alessio Mondini, Virgilio Mattoli, Barbara Mazzolai

**Affiliations:** ^1^Center for Micro-BioRobotics, Istituto Italiano di Tecnologia, Pontedera, Italy

**Keywords:** growth, robots, plant inspiration, self-building robot, self-assembly

## Abstract

This paper provides the very first definition of “growing robots”: a category of robots that imitates biological growth by the incremental addition of material. Although this nomenclature is quite new, the concept of morphological evolution, which is behind growth, has been extensively addressed in engineering and robotics. In fact, the idea of reproducing processes that belong to living systems has always attracted scientists and engineers. The creation of systems that adapt reliably and effectively to the environment with their morphology and control would be beneficial for many different applications, including terrestrial and space exploration or the monitoring of disasters or dangerous environments. Different approaches have been proposed over the years for solving the morphological adaptation of artificial systems, e.g., self-assembly, self-reconfigurability, evolution of virtual creatures, plant inspiration. This work reviews the main milestones in relation to growing robots, starting from the original concept of a self-replicating automaton to the achievements obtained by plant inspiration, which provided an alternative solution to the challenges of creating robots with self-building capabilities. A selection of robots representative of growth functioning is also discussed, grouped by the natural element used as model: molecule, cell, or organism growth-inspired robots. Finally, the historical evolution of growing robots is outlined together with a discussion of the future challenges toward solutions that more faithfully can represent biological growth.

## Introduction

The generation of a biological organism involves three tightly connected processes: growth (changes in mass), remodeling (changes in material properties), and morphogenesis (evolution of the shape) (Taber, 1995). Growth can be interpreted either as a physical process in which there is a permanent increase in mass, typically with body enlargement or elongation, or it can be seen as an abstract process that describes the increase in system complexity (e.g., by the enhancement of knowledge).

Growth involves the cellular activity of both animals and plants; however, the evolution of organisms in these two kingdoms is completely different. Animals have multiple stages of evolution, they grow until maturity, while plants grow indefinitely, mostly for their entire life. Growth in plants is typically restricted to localized areas, called meristematic zones of roots and shoots, whereas animals’ growth zones are distributed all over the body (Soni, 2010). Animal growth is also known as “determinate” growth, since trajectory and asymptotic size are usually genetically defined and environmental influence has a limited impact. Natural selection through evolution has driven the final size and stage of maturity to better satisfy the mechanical constraints imposed by the ecological role of the animal (Sebens, 1987).

Plant growth, on the other hand, is “indeterminate.” Indetermination stems from the continued growth that extends throughout life. Size reflects the complex interdependence between food resources, metabolic needs, and population density (Sebens, 1987). This strong adaptation to environmental conditions, also called plasticity, characterizes the plant kingdom (Sultan, 2000). In addition, plants and animals usually grow at a very different time scale, and plants use growth to move, explore, and colonize the environment; while for animals, growth and locomotion are completely separate functionalities.

The reproduction of processes belonging to living systems within artificial systems including growth has long been the dream of many scientists and engineers (Neumann and Burks, 1966). Turing (1950) was the first mathematician who tried to reproduce a neural learning process in an automaton suggesting that evolution, learning, and growth could be reproduced within a machine. Likewise, Ulam focused on biology as a source of mathematical problems to be modeled, e.g., growth and evolution (Beyer et al., 1985).

Since then, the concept of growth from a variety of biological models has been widely used in computation, for instance, bacterial colony growth has been studied and used for optimization algorithms (Passino, 2002) and robot localization (Gasparri and Prosperi, 2008). Slime mold has been studied as a biological computing device due to its growth and propagation strategies in order to solve optimization or graph-theoretical problems, for example, in the field of transport networks (Tsompanas et al., 2015), for maze-solving computations (Adamatzky, 2015), and in general as a model for sensing and computing (Adamatzky, 2016). L-systems, which are string rewriting systems (Kari et al., 1997), have been introduced to model plant growth, and combined with a neural controller, they have also been used for the simultaneous evolution of the body and brain of virtual creatures (Hornby and Pollack, 2001).

As a physical process, growth in robotics is mainly associated with self-assembly and self-reconfigurability. These are both properties of “modular robotics”—a branch of robotics where a robot is composed of many modules, each with its own actuation and sensing capabilities—where, self-assembly allows modules to physically connect to each other. Self-reconfigurability, on the other hand, is the ability of a modular robot to modify its morphology by rearranging module connections.

The concept of a “growing robot” should not be limited to these two definitions. Regarding the biological meaning, a growing robot should be perceived as a robotic entity that modifies its body structure by the incremental addition of material. By material, we mean any components, modules, or matter that can be supplied (e.g., from a storage) to the robot or that can be found directly in the environment and is connected or incorporated by the robot with a sort of self-building process. Such material not only contributes to the body mass increase and morphological variation, but also enhances the robot with more capacities, e.g., the ability to move, to increase perception by distributed sensing, or in general the ability to accomplish tasks which would otherwise be impossible.

Thus, self-assembly defines a category of robots which, to a certain extent, implement growth functions. These robots, in fact, modify their mass by adding new modules to a main entity, while self-reconfiguration can be considered as an extra feature, not strictly related to growth. In fact, not all reconfigurable robots can be considered as growing robots since reconfiguration can also happen in a closed system without an increase in volume or mass of the robot.

Modular robotics is the main popular approach used by engineers to translate the morphological evolution of biological organisms into artificial systems. A more extensive understanding of biological processes involved in growth mechanisms can help in improving bioinspired approaches to generate artifacts closer to biological growth.

Bioinspiration has already helped to find solutions for the adaptation of robotic bodies and behavior to environmental changes (Pfeifer et al., 2007). The study of neural systems and evolutionary processes has guided heuristic solutions to problems that are too complex for analytical methods and toward the “intellectual” growth of machines (Goldberg and Holland, 1988; Floreano and Mattiussi, 2008). Because of their particular features and difference from animals, plants have already contributed to robotics through, for example, sensing (Lucarotti et al., 2015), materials (Li and Wang, 2016), actuation (Sinibaldi et al., 2014), and control strategies (Sadeghi et al., 2016). Such perspectives have given rise to plant-inspired robotics.

This new field of robotics is attracting interest by roboticists and engineers (Mazzolai et al., 2014; Wahby et al., 2016) and has also brought the concept of plant inspiration to modular robotics. For instance, Soorati and Hamann (2016) mimic plant growth following the traditional approach of modular robots by arranging a set of Kilobots (Rubenstein and Nagpal, 2010) in a branch-like shape according to light distribution.

The utility of a robot that evolves and grows lies in its enormous potential adaptability. In unstructured environments, where constraints are not well known beforehand and where access is limited or not recommended for humans (e.g., after an earthquake), or to locate objects that are difficult to reach, a robotic system capable of quickly adapting its locomotion, morphology, and functionality according to the environment and the task can be much more effective in terms of intervention time and working efficiency.

**Figure 1:**
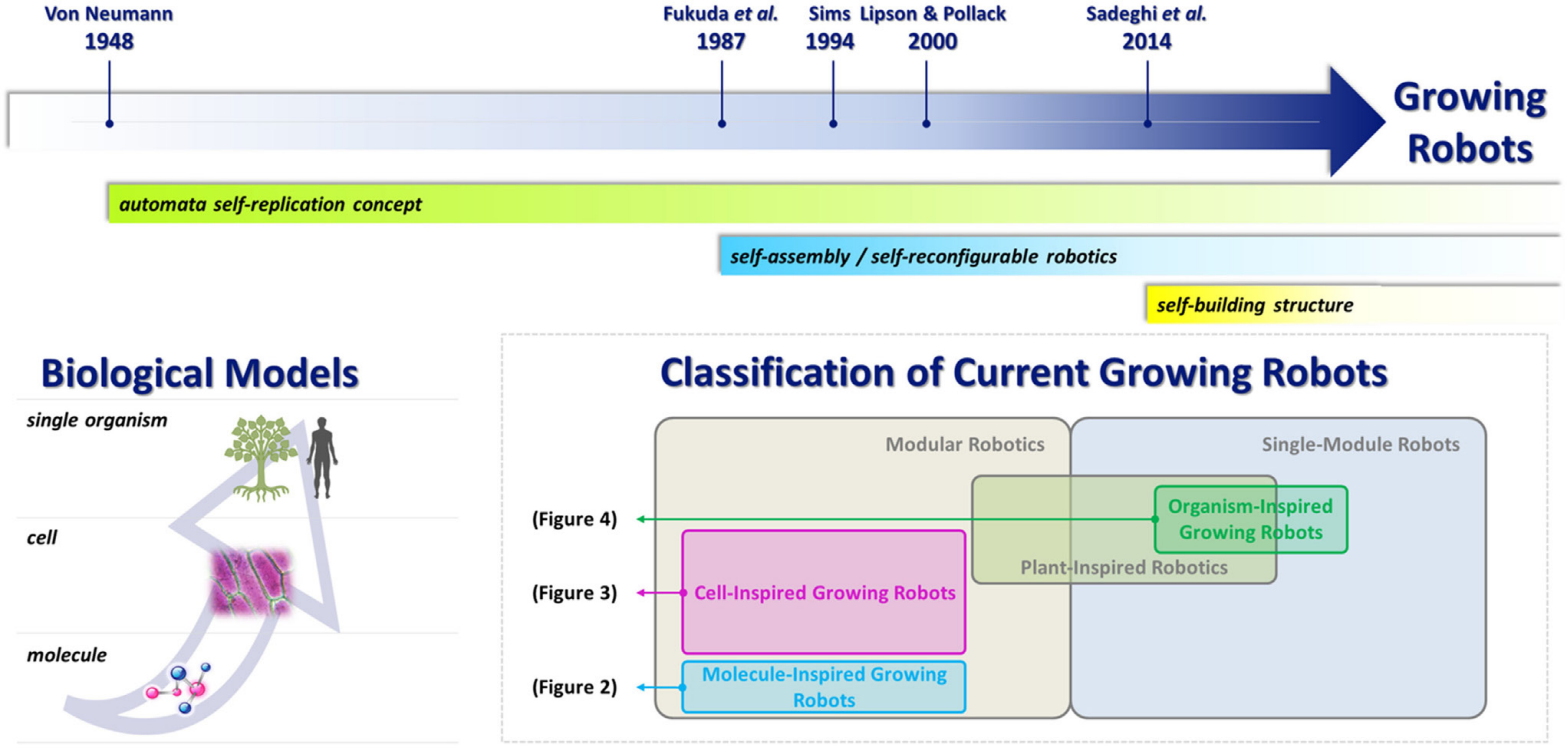
Top: major milestones in relation to growing robots and the concepts developed from the most important contributions. Bottom-left: biological models used for the classification of growing robots. Bottom-right: robotic groups explored in the literature (modular and plant-inspired robotics).

This paper reviews the milestones in the concept of growth in robotics. Section “Toward Growing Robots” focuses on the historical approaches and on the recent results achieved in the area of plant-inspired robotics, specifically on the first robot inspired by roots that move in soil by growing its body. In Section “Bioinspiration Toward Robots that Grow,” we discuss a selection of robotic solutions grouped according to the biological system model and critically analyze the concept of growth implemented. The classification adopted in this paper to categorize growing robots is based on the complexity, or hierarchy, of the natural element addressed: *molecules*, which are the elementary units for life; *cells*, which assemble forming tissues; and an entire *organism* which is composed of the elementary units. Figure 1 gives an overview of this classification. Finally, in Section “Discussion and Conclusion,” we provide a general discussion on the future challenges.

## Toward Growing Robots

The concept of growing machines can be attributed to John Von Neumann, when back in the mid twentieth century he discussed the concepts of self-reproductive automata and “complication.” The “concept of complication” expresses the idea that natural organisms reproduce themselves without decreasing complexity, but instead, through evolutionary processes, new systems are more complex than the parent systems. He speculated if and how such a concept could have been reproduced into automata. Starting from Turing’s theory of computing automata and Ulam’s suggestion about a cellular model (Beyer et al., 1985), Von Neumann (1951) formalized his idea on how an automaton can generate another identical automaton starting from a reservoir of floating elements.

Considering the high level of complexity of natural systems, Von Neumann suggests an approach based on the translation of natural processes into artificial systems by two steps: (I) breaking down the problem into sub-problems, e.g., a single organism is made up of many elementary units; (II) understanding how these individual elements are organized and contribute to the functioning of the whole system.

This idea of decomposing a complex organism into smaller and simpler units was later adopted for the first time in robotics by Fukuda et al. (1988), who presented his concept of a dynamically reconfigurable robotic system (DRRS) implemented in CEBOT (a cell structured robot). This seminal work introduced the concept of a robot not as a single unit but as a distributed robotic system made up of separate autonomous and heterogeneous units, called cells. These robotic cells can be functionalized and are able to communicate with each other, to approach, connect, and disconnect automatically. The composition of cells forms a single module, like a tissue; several modules are assembled to make a single structured robot. The system can adapt its structure to the environment and perform a task by a different combination of modules. By adopting the same strategy of recombination, such a system shows self-repairing and fault tolerance abilities and is able to continue operating even when a failure occurs. The theory at the basis of these decomposed self-assembling robots went far beyond the real implementations—several versions of CEBOT were implemented (Mark I, II, and III)—which, however, were made up of only two modules (Fukuda and Nakagawa, 1988). Despite this, CEBOT was the first prototype of a modular robot, opening up the area of “cellular robotics,” also named “modular robotics.”

Cellular robotics is a broad research area where simple or primitive intelligence (e.g., social insects) are studied and adopted to organize a group or community of robots in terms of their coordination and cooperation (Fukuda and Ueyama, 1994). The main characteristics include: *modularity*, representing cellular structures; *simple functionality*, each cell has limited capabilities; and *decentralized control*, the intelligence of the robot emerges from the interaction among cells. Cellular modules are mainly prefabricated mechatronic cells whose assembly and combination constitutes a single individual (a cellular robot). A community of individuals who interact with each other constitutes a cellular robotic system. Based on this concept, cellular robotics also incorporates the field of “swarm robotics” (Beni and Wang, 1993; Fukuda and Ueyama, 1994).

Modular robotics has made a step toward Von Neumann’s idea of self-reproductive automata possible, through the exploration in robotics of self-organization and self-construction properties adopted by living systems. After Fukuda’s DRRS, the challenge was to obtain a system not limited by the initial design, but which could extend itself through construction methods, and the combination or connection of cells. The dynamic reconfiguration enables the structure, size, and functions of the robot to be adapted according to the task. Self-assembly, self-reconfigurability, and distributed control became the features of many works carried out by the robotic community. Which of these works actually incorporate properties of growth is discussed below.

An alternative approach to cellular robotics was proposed by Lipson and Pollack (2000) with the concept of a continuously self-designing reconfigurable robot. They proposed to combine the power of evolutionary computation to design the body and develop the control at the same time, with additive manufacturing to fabricate it. On the basis of their approach, a single complex robot is composed of three main units: one unit computes the design, the second unit fabricates the body, and the third one is the result of the process, i.e., the body produced. The idea is to produce a robot that without human intervention is able to self-produce control and design of the body that best satisfy a task.

Lipson and Pollack also aimed to develop a robot made of recycled material and components that could be fused and readapted for new morphologies. They implemented an initial idea based on an evolutionary process capable of producing a combination of linear actuators and bars connected with free joints for the body morphology which can travel for a certain distance. In this process, a neural network is evolved for the control and the outcome of the design is then produced through a commercial 3D printer machine. The components need then to be assembled by hand, motors are added, and then programmed. The main limitation of their approach was the technology available. Additive manufacturing equipment seemed impractical as part of a robot and assistance by humans was essential to assemble the components, although the design of the robot was automatically generated by a computation unit. On the other hand, in Lipson’s vision, additive manufacturing can be seen not only as an alternative to classic manufacturing techniques (e.g., molding or assembling), for instance, in the creation of the body of a robot [e.g., Bartlett et al. (2015)], or to assist and enhance robot functionalities [e.g., used to build tools or grippers by the robot itself (Wang et al., 2014a)], but also as an integrated and essential component of the robotic system. It enables unpredictable structures to be created, defined by the control, and to explore designs that would otherwise be potentially difficult to manufacture with classical approaches.

The first methodology to evolve body and control together was proposed by Sims (1994), where a series of different virtual creatures compete in simulated three-dimensional worlds. Evolutionary selection rewards those with the highest scores, letting them survive and reproduce. Morphology and behavior evolve simultaneously. The genotype is represented by directed graphs describing the instructions for the creatures’ growth. A similar approach (tree-based genotype) was later used by Marbach and Ijspeert (2004) in their Adam simulator which co-evolved configuration and a PD control for homogenous chain-type modular robots. Their objective was to develop a simulator to define the configuration of physically implementable modular robots with a fixed and known design, together with the control for the appropriate locomotion of the defined structure. von Haller et al. (2005) co-evolved the body configuration of an underwater modular robot with a neural network controller based on a central pattern generator.

Another perspective of the evolution of virtual creatures is to provide an abstraction from a real robot (Bongard, 2013). This approach converged into “evolutionary robotics.” The main goal of this methodology is to reproduce evolutionary processes to find the optimal robotic structures for a specific task that do not necessarily resemble existing creatures. This field of robotics also exploits the “evolution of development,” which means the process by which a creature evolves from the embryonic stage to maturity, while being affected by evolution over time (Bongard, 2013). Bongard (2011) proposed the evolution of an anguilliform virtual creature that mutates into a legged robot in the early stage of evolution. Locomotion gaits evolved with morphological changes, and he observed that creatures evolving from anguilliform to legged creatures performed the transition more rapidly with gaits that were also more robust compared to creatures that already had legged bodies.

Evolutionary robotics also represents a biological instrument for understanding morphogenetic properties and subsequently the evolutionary transitions of real creatures. Evolutionary robotics has thus been included in the broader field of “morphogenetic engineering” (Doursat et al., 2012), which is an evolution of cellular robotics since it is based on a deeper understanding of how organisms and populations reliably accomplish morphogenetic tasks. It also examines how self-organization capabilities can be translated into engineered systems and which principles can achieve a morphogenetic system. The basic principles are still the same as cellular robotics: *modularity* and *decentralized control*; although it is endowed with advanced tools and algorithmic approaches, such as those offered by evolutionary robotics. These approaches simultaneously explore the design and control of autonomous systems, which are capable of developing complex and heterogeneous morphologies and functions in a decentralized manner.

The results in this field have demonstrated the feasibility of fabricating an evolved modular creature (Hiller and Lipson, 2012) and the transferability of an evolved control onto a physical not evolving robot (Koos et al., 2013), putting significant effort in the computation of the robot design and morphological evolution in simulation. The approach adopted by evolutionary robotics has also been used for the evolution of virtual soft bodies (Joachimczak and Wróbel, 2012), especially for the investigation of material properties and their effects on robot behavior (Rieffel et al., 2014; Corucci et al., 2016). All of these works, and many others, demonstrate the feasibility of obtaining an intellectually growing and adaptable robot. However, the step from simulation to the physical creation of a growing—not only intellectually but also morphologically—completely autonomous working robot is still ongoing. Again, the main challenge seems to lie in the physical transduction of evolutionary processes for the morphological evolution and growth of a real robot.

A step further toward the physical implementation of robots that grow has been achieved by taking inspiration from the concept of growth in plants (see Organism-Inspired Robots). The starting approach originated from the observation of the high adaptability of plants to harsh environments and their ability to colonize soil, skills that can be translated into artificial devices for soil penetration and exploration (Mazzolai et al., 2008). By investigating different strategies adopted by plants, a series of energetically efficient technological solutions to movement in soil have been proposed. For instance, in Sadeghi et al. (2013), the release of dead cells from the root tip was implemented with a mechanism of skin eversion from the inside of a tubular shaft to the outside. This was done by creating an interface between the shaft and the soil and demonstrated the reduction in friction perception in soil penetration. In Del Dottore et al. (2016), the oscillatory movement that is also actuated at the tip level in plant roots was used in a robotic root to demonstrate the reduction in forces needed to penetrate into soil with respect to a straight penetration. The particular features of roots and shoots that enable them to grow from their apical regions by cell division and elongation were first translated into a root-like device that deposits a filament in a circular manner, preserving the contact of the filament with the artificial tip, thus providing sufficient force for pushing the probe into soil (Sadeghi et al., 2014). Sadeghi’s study paved the way for a new generation of robots inspired by plants which also provides an alternative to cellular robotics aimed at physically implementing the concept of the “self-building” robot.

## Bioinspiration toward Robots that Grow

This section provides an overview of the robots that have been inspired by the mechanisms of growth implemented by biological systems and in which a bonding strategy has led to this feature being physically implemented. The main categories are modular robots, with physical self-assembling capabilities, and plant-inspired robotics where robots that grow imitate the strategy adopted by plants of growing from the tip.

We have categorized the selected robots according to an increasing hierarchical dimension of natural elements, starting from molecules up to a single complete organism (see Figure 1; Table 1).

**Table 1:** Growing robots.

Environment	Max.growth	Dimension (cm)	Properties	Binding mechanism	Energy	Role of growth	Dev. level	Task
**MOLECULE-INSPIRED ROBOTS**								
**Griffith, 2004; Griffith et al., 2005—random parts**								
Floating table	30 modules	5 × 5 × 1.5	SA; SR	McE	OB	Replication	PoC	Structure

**White et al., 2004—stochastic self-reconfigurable robots 2D—Figure 2A**								
Floating table	3 modules	6 × 6 base	SA; SC	Em	EP	Assembly	PoC	Structure

**White et al., 2005—stochastic self-reconfigurable robots 3D—Figure 2B**								
Tank with fluid	2 modules	10 × 10 × 10	SA; SC	Em; Ff	EP	Assembly	PoC	Structure

**Haghighat et al., 2016—lily robots—Figure 2C**								
Fluidic surface	6 modules	5 × 5 × 2.5	SA	Em	OB	Assembly	PoC	Structure
**CELL-INSPIRED ROBOTS**								
**Fukuda and Nakagawa, 1988; Fukuda and Nakagawa, 1990—CEBOT (Mark II)—Figure 3A**								
Ground	2 modules	9 × 18 × 5	SA	McU	EP	Assembly	Partial^a^	Manipulation

**Yim et al., 2000; Yim et al., 2002—PolyBot—Figure 3B**								
Ground	6 modules arm adding 1 module	6 × 7 × 11	SA; SC	McPHsma	EP	Assembly	Operative	Locomotion

**Payne et al., 2004; Rubenstein et al., 2004—CONRO—Figure 3C**								
Ground	2 modules^b^	10.8 × 4.4 × 4.5	SA; SC	McPHsma	OB	Assembly	Operative	Locomotion

**Murata et al., 2006; Kurokawa et al., 2008—M-TRAN—Figure 3D**								
Ground	17 stationary modules + 3 moving modules	6.5 × 13 × 6.5	SA; SC	McU	OB	Assembly	Operative	Locomotion

**Yim et al., 2007—CKbot^c^—Figure 3E**								
Ground	3 clusters of 5 modules	Not specified	SA; SC	Mg	OB	Assembly	Operative	Locomotion

**Wei et al., 2011—Sambot—Figure 3F**								
Ground	2 modules	8 × 8 × 10.2	SA; SC	McU	OB	Assembly	Operative	Locomotion

**Davey et al., 2012—SMORES—Figure 3G**								
Ground	2 modules	10 × 10 × 9	SA; SC	Mg	OB	Assembly	Partial^d^	Locomotion

**Romanishin et al., 2013—M-Blocks—Figure 3H**								
Ground	1 module assembles to a group	5 × 5 × 5	SA; SC	Mg	OB	Assembly	Operative^e^	Locomotion

**Qiao et al., 2014—modular self-reconfigurable robot—Figure 3I**								
Ground	4 modules^f^	26.4 × 7.1 × 6.5	SA; SC	McPH	OB	Assembly	Operative	Locomotion

**Spröwitz et al., 2014—Roombots—Figure 3J**								
Ground	4 modules^g^	11 × 11 × 22	SA; SC	McU	OB	Assembly	Operative	Furniture building
**ORGANISM-INSPIRED ROBOTS**								
**Sadeghi et al., 2017—plant-root-like robot growing with 3D printing—Figure 4A**
Subsoil	140 mm	5Ø × 6.3L^h^	SB	FDM	EP	Locomotion	Operative	Soil penetration in multi directions

**Hawkes et al., 2017—soft robot growing with inflation mechanism—Figure 4B**								
Ground/air	70 m	4.8Ø^i^	E	IM	EP	Locomotion	Operative	Ground navigation

*Environment represents the operative location of the robot. Max. growth is the maximal growth shown in papers; it represents the maximal number of modules performing assembling or the maximal length of elongation. Dimension is the single module size. Properties: SA, self-assembly; SR, self-replication; SC, self-reconfiguration; SB, self-building structure; E, extension. Binding mechanism: McE, mechanical latch, regulated electromagnetically; McU, mechanical hooks; Em, electromagnets; Mg, magnets; Ff, pressure of fluid flow; McPHsma, mechanical pin-hole and SMA; McPH, pin-hole connector; LL, alignment of layer by layer filament; FDM, fused deposition modeling; IM, inflation mechanism. Energy: OB, on board; EP, external power. Role of growth defines for which functionality a growing process is used. Level of development of the system with respect to the growing process: simple proof of concept (PoC) of growth, partial, or operative. Task describes for which operation the robot has been developed (*Structure* means structure formation)*.

*^a^The system was modeled for manipulation tasks in industrial environments. The authors achieved the deployment of three modules demonstrating self-assembly with two. Manipulation was not demonstrated*.

*^b^Modules need to stay in the proximal distance*.

*^c^An evolution of the earlier PolyBot robot*.

*^d^The authors show self-assembling, self-reconfiguration, and lifting of a module with two modules*.

*^e^Crawling is achieved for a group of modules*.

*^f^Self-assembling is essential for reconfiguration, the authors show the assembling of 1 module and then of 2 distinct modules to a cluster of 2 modules*.

*^g^The authors show the assembling of 2 clusters of 2 modules to overcome a convex edge module*.

*^h^Length of the apical module*.

*^i^Diameter used to obtain the maximal length of 70 m*.

### Molecule-Inspired Robots

**Figure 2:**
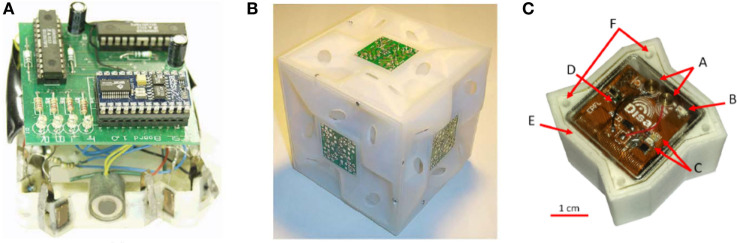
Molecule-inspired robots. **(A)** the square implementation of White’s robots assembling in 2D space (White et al., 2004) (photo courtesy of H. Lipson, Columbia University); **(B)** the module with the fluid flow bonding mechanism is presented as a sample for the White’s robots assembling in 3D space (White et al., 2005) (photo courtesy of H. Lipson, Columbia University); **(C)** a single module of Lily robots (© [2018] IEEE. Reprinted, with permission, from Haghighat et al., 2015).

This category includes robots inspired by molecule polymerization in DNA formation (Figure 2). This process is the basis of all evolutionary processes, including growth. The artificial implementations are based on stochastic collisions of particles—Brownian motion (Einstein, 1956)—which are not active when disconnected but acquire functionalization once a bond is established, leading to the ability to assemble, seen here as growth.

One of the first examples comes from Griffith who fabricated small entities capable of self-assembly (Griffith, 2004). His goal was to reproduce the assembling capabilities of biological systems at the nano/micro-scale in order to devise new fabrication technologies that would enable self-assembly three-dimensional structures to be created that would perform error-prevention, error-correction, logic, self-replication, and self-repair by the replication of part assemblies. The result of his work was a self-replicating system composed of a finite-state machine randomly moving on a floating table which could physically connect or disconnect with/from each other in response to neighboring communications (Griffith et al., 2005). In this implementation, each part does not need to store a copy of the entire structure but queries neighbors in order to test self-similarity and its position in the growing replicant. These machines were able to compose strings in 2D—sequence of variable numbers of modules only in one line—and to create copies of these strings. The growing entity is a single string, which starts from a single module and increases in size and functionality thanks to the assembly of other modules.

In White et al. (2004), an approach based on passive modules moving on a floating table that bind stochastically was proposed. White proposed two geometries, a square and a triangle, for 2D motion and assembly. Binding starts from a single active module (a seed) which, according to internal rules, can activate or deactivate bonding sites. The motion is based on statistical mechanics and on attraction properties of active bonding sites. Once a bond is established, the resulting structure is then able to activate global and distributed sensing, actuation, and computation. Bonding sites are implemented with electromagnets and the structure starts to grow from a seed module. Two 3D versions of the same principle have also been implemented (White et al., 2005): one implementation uses magnets for bonding, as in the 2D case; the second implementation instead uses the activation or deactivation of internal pumps to form or detach bonds. In both 3D implementations, modules passively move in a liquid which through agitation introduces randomness into the system. A module fixed at the bottom of the tank acts as a seed and attracts the floating modules.

The same floating principle was proposed in Haghighat et al. (2015, 2016), where Lily robots assemble themselves following graph grammar rules, which specify conditions of bonding when randomly mating according to a target structure. These robots can be configured into different shapes.

The Programmable Parts proposed in Bishop et al. (2005) are triangular robots that passively float on an air table and bind to each other upon random collisions. Chemical reactions are imitated by graph grammar rules employed to establish or detach a bond. However, the stochasticity and assembling capability are insufficient to make it a growing robot. In fact, it is only able to assemble itself in a single form which is strictly dependent on the geometry of the module. The combination of a different morphology with the stochasticity of the environment has been demonstrated to affect the clusterization of modules enormously (Miyashita et al., 2011). In addition, the simple effect of morphology has been analyzed, for instance, in Miyashita et al. (2009) or Nakajima et al. (2012), also demonstrating in these cases the different clusterization according to different geometries.

Molecule-inspired robots can artificially implement a natural process without performing complex tasks, although they are extremely dependent on the module geometry that defines the assembly and on the floating environment that provides the energy for locomotion. They are thus mainly limited to PoCs.

### Cell-Inspired Robots

This category potentially includes all modular robots, since they derive from the idea of replicating cell structures. Primarily, ideologically they resemble cells; however, they do not implement the biological functionality of growth by cell division and self-production.

**Figure 3:**
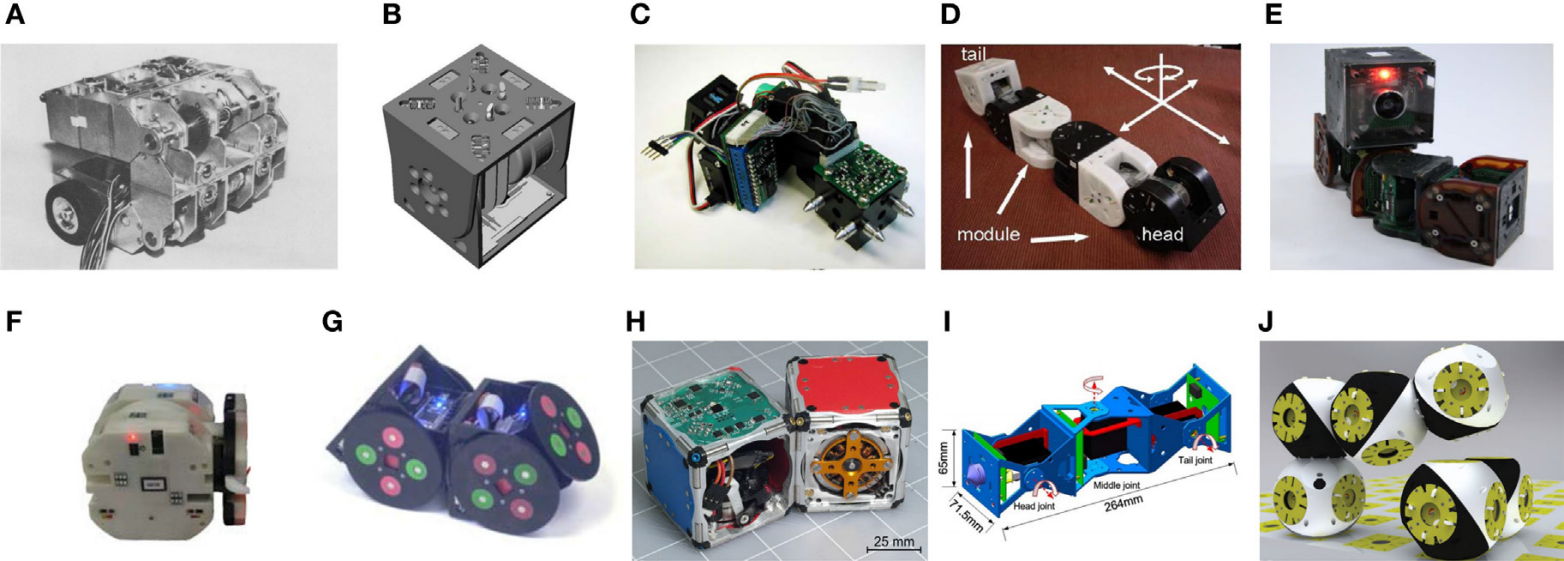
Cell-inspired robots. **(A)** The Mark II implementation of CEBOT [Reprinted by permission from Springer Nature Terms and Conditions for RightsLink Permissions Springer Customer Service Centre GmbH: Springer Nature, Journal of Intelligent and Robotic Systems (Fukuda and Nakagawa, 1990), CC BY (1990)]; **(B)** rendering of a single module of PolyBot model G3 (© [2018] IEEE. Reprinted, with permission, from Yim et al., 2002); **(C)** a single module of CONRO robot (Payne et al., 2004) (Reprinted by permission from Rubenstein, Payne, Will, Shen/USC, ISI); **(D)** a cluster of modules approaching a docking station cluster of M-TRAN robot (© [2018] IEEE. Reprinted, with permission, from Murata et al., 2006); **(E)** a cluster of modules of CKbot with camera module on top (© [2018] IEEE. Reprinted, with permission, from Yim et al., 2007); **(F)** a single module of Sambot (© [2018] IEEE. Reprinted, with permission, from Wei et al., 2011); **(G)** two modules of SMORES (© [2018] IEEE. Reprinted, with permission, from Davey et al., 2012); **(H)** two modules of M-Blocks (© [2018] IEEE. Reprinted, with permission, from Romanishin et al., 2013); **(I)** a single module of the modular self-reconfigurable robot presented in Qiao et al. (2014) (Reprinted with permission from License Attribution 3.0 Unported (CC BY 3.0)); **(J)** Roombots: two clusters composed of two modules connected to a grid during assembly (© [2018] IEEE. Reprinted, with permission, from Spröwitz et al., 2010).

There are several reviews on modular robots with self-assembling and self-reconfigurable capabilities (Gilpin and Rus, 2010) (Groß and Dorigo, 2008) (Yim et al., 2009). Modular robots are usually classified depending on the architecture (lattice, chain, mobile, and hybrid) or method of reconfiguration (deterministic, stochastic). Our interest is in the growth functionality that these robots implement. Our aim is not to provide another review on modular robotics but to select only those modular robots that show growing capabilities by means of self-assembly, which for instance purely lattice or chain modular robots do not have in general (Figure 3).

However, in terms of modular robotics, the focus has been on defining strategies for system reconfiguration, which uses the concept of swarm intelligence to provide more independence to every single module and to create strategies for collaboration among modules. In addition, fabrication technologies have been aimed at achieving mobile autonomous modules that physically connect with each other. Such systems, which mostly move and connect forming 2D configurations [e.g., Swarm-Bot (O’Grady et al., 2005), ULGEN (Ercan and Boyraz, 2016), X-CELLs robot (Hong et al., 2011)], are beyond the scope of the present review. This is because they mainly simulate the collective behavior of colonies of individuals, where a single individual already has an enhanced level of autonomy and the assembly does not provide a real augmentation of different capabilities. As mentioned in Section “Toward Growing Robots,” these works in swarm robotics resemble cellular robotic systems more than cellular robots.

CEBOT (Fukuda and Nakagawa, 1988), which was built for manipulation purposes in industrial environments, is thus the precursor of modularity and distributed control (see Toward Growing Robots) although it did not achieve full functionality. CONRO (Rubenstein et al., 2004) shows its growing capability by the self-assembly of two segments, composed of two modules each. Segment docking needs to be performed with the appropriate alignment of male-female mechanisms and the detection of the two segments is constrained by IR interface alignment. The PolyBot (Yim et al., 2002) assembly strategy was designed very similarly to CONRO, which in addition to CONRO shows the docking of a six-module arm to a single module positioned at a fixed and known location.

Its more advanced successor CKbot (Yim et al., 2007) uses visual feedback to locate neighboring disconnected modules. Each disconnected cluster of modules needs a camera module to guide its docking, and all clusters search for the others. Visual feedback had already been used by M-TRAN (Murata et al., 2006), where a single camera module is positioned on a stationary cluster to calculate the position and orientation of the disconnected cluster of modules and guides them toward the docking position.

SMORES (Davey et al., 2012) is a hybrid system which can connect two modules by magnets closely positioned to autonomously induce the connection (search and approach are not described). M-Blocks (Romanishin et al., 2013) are independent robotic blocks which assemble by magnetic edges and faces, using a unidirectional reaction wheel (flywheel) to create an approximate impulse of torque. When a block is not aligned with a goal position, the module actuator will use high torque leading to a random movement; instead, if the module is aligned with the goal position, torque is used, leading to a controlled rotation toward the goal. Qiao et al. (2014) show how a mobile module docks itself to a module fixed at a defined position.

Wei et al. (2011) present another good example of cell-inspired growing robot (Sambot), although it probably lies on the boundary between swarm robot and a self-assembling mobile robot. Through self-assembly, this multi-robot system can form a variety of robotic structures with locomotion capabilities. Docking is achieved with IR interfaces and mechanical hooks. Mechanical hooks are also used by Roombot modules (Spröwitz et al., 2014) to connect to each other. Spröwitz shows the ability of the two modules to connect in order to pass a convex edge; however, modules need a pre-computed set of steps and also need to be connected to a grid with passive connectors embedded in the floor.

In cellular robotics, most of the focus has been on docking strategies, for searching, approaching, and connecting disconnected modules. To obtain a bonding mechanism, a module needs to apply a sufficient force to connect to another one and hopefully lift it. At the same time, a fast release is required. In addition, strategies for finding the optimal reconfiguration are important. Generally, search and step planning algorithms are implemented on a separate working station and only then are the steps to follow are provided to each module.

Growth, or in this case self-assembly functionality, is not usually a key objective in modular robotics. One example where growth acquires more relevance is the Proteo robot (Bojinov et al., 2000). Proteo is a homogeneous multiagent self-reconfigurable robotic system, which according to the authors, is able to grow stable structures. Bojinov configured the robot using local attractors—modules called seeds—which produce a gradient of attraction toward the desired global configuration, like chemical gradients guiding migrating cells (Meinhardt, 1993). However, the concept of growth is used in Proteo as a strategy—the directed evolution of the shape—not for the assembly but for reconfiguration, to change the robot shape from one configuration to another. Moreover, Proteo has shown its capabilities in simulation and not in a physical implementation, and it does not implement a self-assembling or alternatively self-building strategy.

All the cases reported in this section have constraints in successfully connecting the modules. In fact, one of the two disjoint components needs to be fixed to a defined position; or both components need to contribute to the assembling process, making it difficult to recognize the actual entity increasing in size, or rather, growing. Moreover, the granularity of growth cannot be tuned: the minimal size is predefined by the module size (discrete process). The assembly is clearly not real growth, but it has been the closest approximation to growth and successful artificial process for several years.

### Organism-Inspired Robots

This category considers robots inspired by the growth strategies adopted by an entire organism, either simple or complex, such as animals, fungi, and plants. Unlike cell-inspired growing robots, where several modules connect to each other to emulate the assembly of cells, organism-inspired robots include single-module systems which are able to incrementally add material in specific areas of their body.

**Figure 4:**
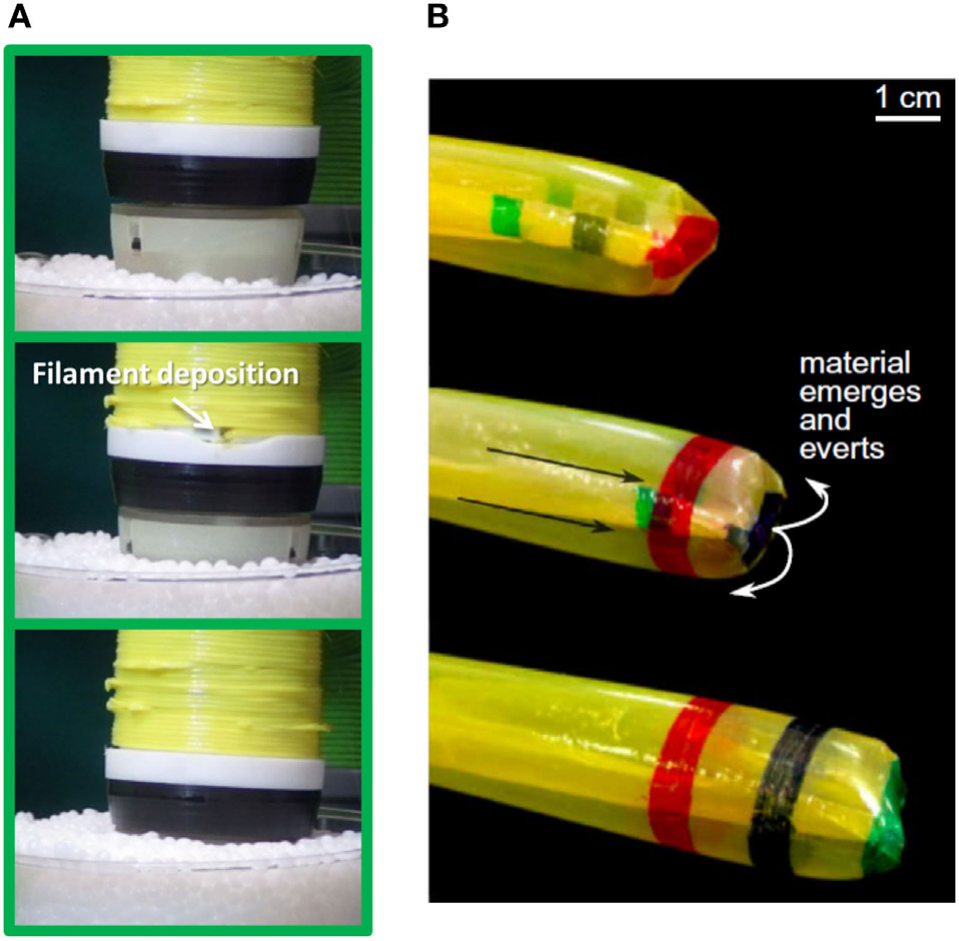
Organism-inspired robots. **(A)** The first implementation of the growing robot inspired by plant roots, with a thermoplastic material used to build the structure with additive manufacturing (Sadeghi et al., 2017); **(B)** Body elongation sequence of the soft robot with skin eversion induced by pressurization (From Hawkes et al., 2017. Reprinted with permission from AAAS).

Sadeghi et al. (2014) proposed the first device capable of vertically penetrating soil through an inert filament at the tip, thus imitating growth from the tip adopted by plants. The subsequent result of this pioneering work was the creation of a plant-root-like robot able to build its own body structure (Sadeghi et al., 2017) (Figure 4A). The robotic root hosts a miniature 3D printer inside its tip and uses a thermoplastic filament to build a stable hollow body where layers of fused material adhere to each other and solidify consolidating the structure (FDM—fused deposition modeling approach). System bending is achieved by differential growing, by depositing different amounts of raw material at the opposite sides of the robot. The tip embeds sensors and a control unit with a bioinspired behavior (Sadeghi et al., 2016), which drives the direction of growth actuated on the back of the tip allowing the following of attractors or avoidance of repellents. This work represents the first physical solution to self-creating robots, by integrating additive manufacturing techniques inside the robotic bodies.

The adoption of thermoplastic material has also been proposed by Wang et al. (2014b) who developed a spider-like robot. In this case, the material is released by the robot for the creation of a dragline. Yet, the continuous filament released by this robot cannot be considered as an integral part of the robot’s body since it is an external component used by the same robot for its locomotion—by lateral wheels sliding on the formed filament—not to morphologically change the body, thus invalidating the function of growth.

Hawkes et al. (2017) exploited the idea of body elongation adopted by several biological systems such as fungal hyphae, neurons, or pollen tubes in seed plants. Hawkes et al. implemented a robotic system that can elongate its body from the tip by skin eversion using pressurized chambers, and by individually controlling pressurization on each chamber, the system can bend for aboveground environment navigation driven by a visual processing controller Figure 4B. In fact, there are fixed pinch-latch mechanisms along the skin, with which higher pressure unlatch, thus enabling bending on the opposite side.

In the above examples, the main limitation lies in the quantity of material available. This means that the ability to grow is still dependent on and limited by a reservoir of material that needs to be provided to the entity managing deposition or inflation.

## Discussion and Conclusion

This paper presents the historical evolution of growing robots. We propose a definition of this category where robots physically evolve their artificial bodies by the addition of material. This principle represents a dream for many scientists and engineers, and different approaches have long been proposed in robotics. Ideally, from a long-term perspective, growing robots should be able to self-build their own structure and upgrade functionalities on the basis of elements present in the environment, making them completely independent from a reservoir of material or human intervention.

Currently, none of the reported solutions exploit the environment, however cellular robotics, with modular robots that are capable of self-assembly (modules are able to autonomously connect to each other) represents the first attempt toward this goal. This research has led to the mechanical improvement of physical connections and to algorithmic approaches for template matching, configuration selection or path planning, to reduce the reconfiguration time and optimize module arrangements for a specific task. The concept of growth has been extended to a more general morphological evolution and is typically treated in these robots with the self-assembly and rearrangement of modules.

Taking inspiration from nature, the elongation of tissue has been imitated by everting artificial skin from the inside to the outside of a tubular body (Sadeghi et al., 2013) or by pressurized chambers (Hawkes et al., 2017), obtaining rapid growth and bending capabilities. A new approach has also been discussed that led to the first robot beyond modular robotics capable of building its own structure from a supplied thermoplastic material (Sadeghi et al., 2017). The robot is able to manipulate, change material properties, and deposit the material in order to build a structure that can be considered as a channel for communication, which would be particularly useful in rescue applications, for providing oxygen, water, to pass through other robots, cameras, or sensors. The tubular built structure also easily provides a passage for the delivery of new raw material and energy necessary for the growing process.

In the examples reported as organism-inspired robots, there is a finer granularity of growth compared to the robots in molecule- and cell-inspired robots. Instead of having the minimum growth dictated by the size of a module, organism-like growing robots are able to tune their growth by speeding filament deposition (Sadeghi et al., 2017) or chamber inflation (Hawkes et al., 2017) up or down, sparing the need of technically complex and expensive modules in imitating the growth process.

The mechanisms proposed in this category have various pros and cons. For instance, the exploitation of additive manufacturing in plant-growth-inspired robots enables the exploration of soil and unstructured environments besides the aboveground scenario. On the other hand, an inflating mechanism, for instance, can obtain a faster growth velocity respect to the FDM technique, enabling the robot for a fast reaction in aboveground environment. In fact, in adopting a plotting strategy for material deposition, the process can be quite slow, which can be overcome for instance, by changing the printing technique, e.g., the projection of an entire area, or using a material with a different bonding mechanism, e.g., a chemical bond as in hydrogels. However, with respect to fast growth, a slow process can be more beneficial where high impedance—e.g., in soil—or the high risk of damage—e.g., unstable structures, or human’s tissues—are constraints of the environment where the robot has to move.

Yet, the physical implementation of growing robots is limited by the current difficulties in technologically imitating cell division and absorption of environmental resources.

Self-healing for instance is a feature embedded in natural tissue enabling the reconstruction of dead or damaged tissue, and is also a desirable feature for growing robots. Self-healing properties, which are often confused with the more practical concept of “self-repair” (i.e., maintenance) and implemented in robotics with redundancy and reconfiguration (Murata et al., 2001), have been recently explored in materials (Wool, 2008; Hager et al., 2010; Yang and Urban, 2013). Self-healing polymers have already been adopted in soft robotics as soft pneumatic actuators—e.g., artificial muscles—and soft hands able to re-establish cross-link bonding through a thermoreversible Diels–Alder reaction after being damaged by sharped objects (Terryn et al., 2017). Also, studies are currently being performed to imitate material micro-structures for the dynamic adaptation of the shape, for example using hydrogels (Gladman et al., 2016).

Tissue engineering and synthetic biology (Cheng and Lu, 2012) could provide a complementary approach to classic engineering. In fact, in synthetic biology, the objective is to build new biological systems starting (I) from an existing system and by reducing its complexity (top-down approach)—designing, synthesizing or recombining DNA to preserve essential and desired functionalities (Gibson et al., 2008, 2010)—or (II) building a new system from basic units (bottom-up approach) (Schwille, 2011). This approach has led to the construction of *in vitro* circuits from synthetic DNA switches (Gardner et al., 2000; Kim et al., 2005, 2006), synthetic polymer scaffolds for the development of artificial bones (Song et al., 2004) or actuators powered by biohybrid materials synthesized from natural muscles (Feinberg et al., 2007). This thus demonstrates that synthetic biology offers functional tools for artificial self-assembling and self-replicating systems. The combination of these tools with artificial tissues, functionalization, self-healing properties, and self-building robot concepts could represent the right approach for a new generation of growing robots.

Growing robots can be particularly useful in unstructured environments, especially for search and rescue applications where the tasks and constraints of the environment are not known beforehand. From a long-term perspective, it is possible to envisage a robot that could be released in its minimal configuration and then, with materials available in the environment, could build itself: it could increase and adapt its structure to accomplish the required tasks. This would particularly benefit space and exploratory applications where growing robots would occupy less space. In addition in terms of costs, it would be a much cheaper solution to send a single unit that is able to self-build its structure than a set of modules.

## Author Contributions

ED researched the literature, discussed and wrote the paper; AS, AM, VM, and BM discussed and wrote the paper.

## Conflict of Interest Statement

The authors declare that the research was conducted in the absence of any commercial or financial relationships that could be construed as a potential conflict of interest.
